# Supplementary material for: scATAC-pro: a comprehensive workbench for single-cell chromatin accessibility sequencing data
Source: Genome Biol. 2020 Apr 20;21:94. doi: 10.1186/s13059-020-02008-0 (PMC7169039; doi:10.1186/s13059-020-02008-0)

Supplementary Figures

Fig S1. Effect of data binarization on clustering performance using simulated data with different levels of noise. Data was simulated using a binomial distribution of read counts in peaks. Noise level was controlled using the parameter q. q = 0 indicates no noise while q = 1 indicates the highest level of noise. See methods for details. cisTopic and LSI can only use binarized data. The rest of the methods use non-binarized data by default although they can also use binarized data. **(A)** Adjusted rand index (ARI) for different methods, using the FACS-sorted cell types as the ground truth. **(B, C)** Performance of the same clustering algorithm using binarized and non-binarized data. ARI of Louvain clustering algorithm **(B)** and K-means algorithm **(C)** using 30 principal components calculated on different numbers of variable peaks.

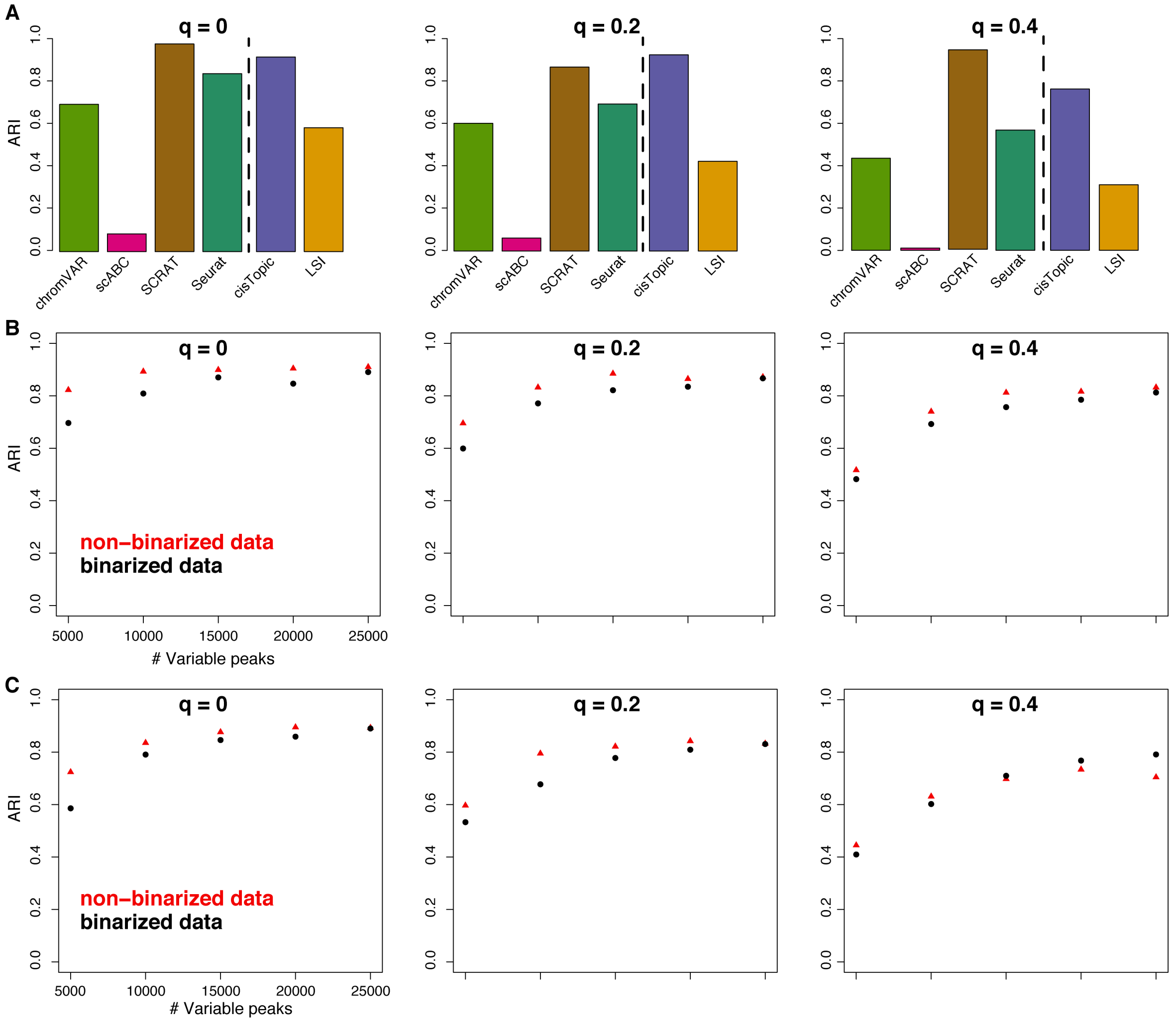

Fig S2. Performance comparison of different clustering algorithms of existing tools using simulated and real data. Simulated data was generated by subsampling aligned reads directly from bulk ATAC-seq data and no noise was introduced. **(A)** Adjusted rand index (ARI) for different algorithms using the FACS-sorted cell types as the ground truth. Cells of each type were subsampled with equal probability from a bulk ATAC-seq data set. **(B)** Computation time of each method. **(C)** Adjusted rand index of 100 sets of simulated data. Cells were sampled from the 13 types with different proportions. The proportions of different cell types were generated based on the Dirichlet distribution (with shape parameter alpha = 3 for each component). **(D)** Adjusted rand index for different algorithms using a real single-cell ATAC-seq data set (Buenrostro2018).

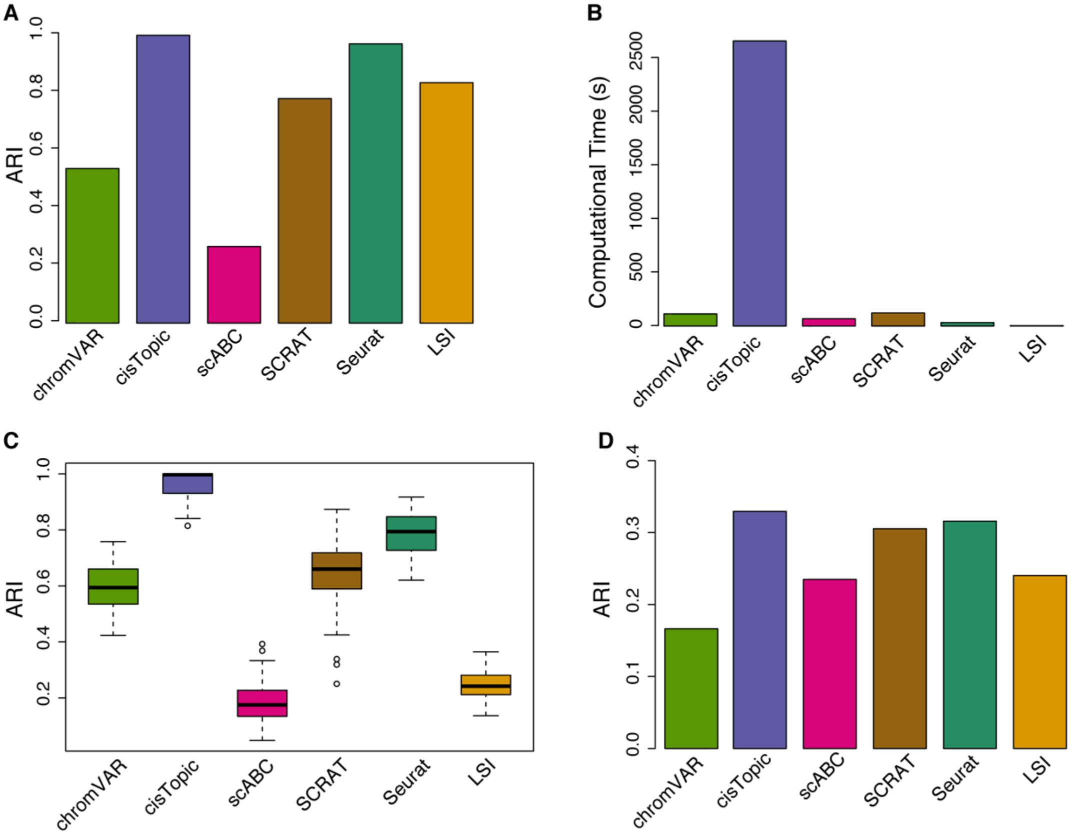

Fig S3. Performance comparison of principal component analysis (PCA) implemented in Seurat and scATAC-pro (Seurat_correct). (A) Computation time as a function of the fraction of features (peaks) used. **(B)** Similarity of the clustering results based on PCA by Seurat and scATAC-pro. Clustering was done using the Louvain algorithm. Similarity was measured using the adjusted rand index.

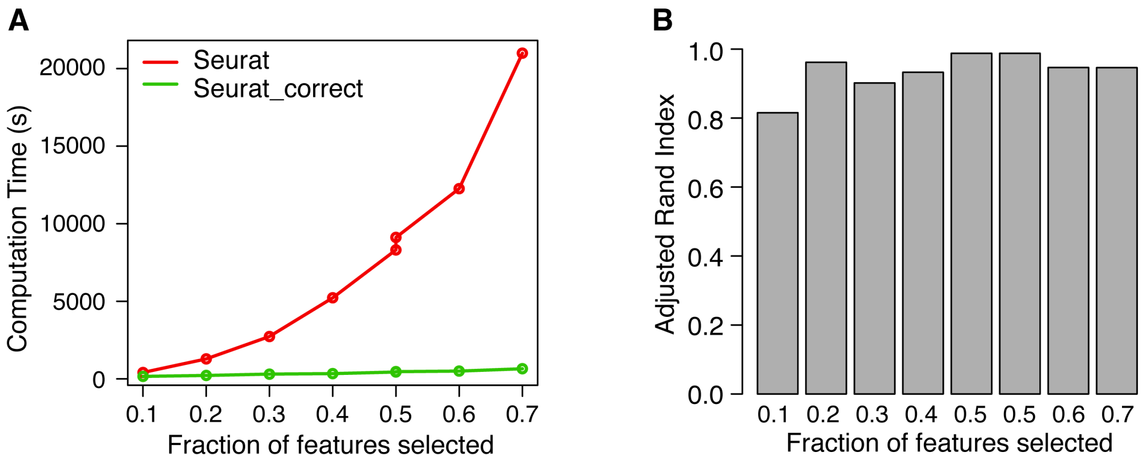

Fig S4. Summary statistics for read mapping, library complexity, and cell calling of Buenrostro 2018 data set. Global mapping statistics are based on all data (**A**). Cell barcode mapping statistics are based on called cells (**B, C**). MAPQ, mapping quality score.

scATAC-pro Report | Global QC | Cell Barcode QC | Downstream Analysis

A Global mapping statistics

Sample: Buenrostro2018

Total_pairs	721497044	100%
Total_pairs_mapped	415044825	57.5%
Total_uniq_mapped	370747998	51.4%
Total_mito_mapped	191278321	26.5%
Total_dups	504435953	69.9%
Total_pairs_MAPQ30	353736920	49%
Total_mito_MAPQ30	163164746	22.6%
Total_dups_MAPQ30	309051781	42.8%
Library complexity		19.5%

B Cell barcode summary

Cell called by	FILTER
Estimated number of cells	2065
Median fragments per cell	24362
Fraction of mapped reads in cells	40.7%
Fraction of MAPQ30 in cells	74.3%

C Cell barcode mapping statistics

Total_pairs	420565191	100%
Total_pairs_mapped	168966302	40.2%
Total_uniq_mapped	265627443	63.2%
Total_mito_mapped	89299919	21.2%
Total_dups	267297950	63.6%
Total_pairs_MAPQ30	144113853	34.3%
Total_mito_MAPQ30	76675376	18.2%
Total_dups_MAPQ30	127675320	30.4%
Library complexity		19.6%

Fig S5. Summary statistics for read mapping, library complexity, and cell calling of Cusanovich 2018 data set. Global mapping statistics are based on all data (**A**). Cell barcode mapping statistics are based on called cells (**B, C**). MAPQ, mapping quality score. Note that this data was processed using a downloaded bam file since the barcode index file is not publicly available.

scATAC-pro Report | Global QC | Cell Barcode QC | Downstream Analysis

A Global mapping statistics

Sample: Cusanovich2018

Total_pairs	149024225	100%
Total_pairs_mapped	149024225	100%
Total_uniq_mapped	149022861	100%
Total_mito_mapped	0	0%
Total_dups	2693797	1.8%
Total_pairs_MAPQ30	134562544	90.3%
Total_mito_MAPQ30	0	0%
Total_dups_MAPQ30	1654782	1.1%
Library complexity		100%

B Cell barcode summary

Cell called by	FILTER
Estimated number of cells	12336
Median fragments per cell	4909
Fraction of mapped reads in cells	70.6%
Fraction of MAPQ30 in cells	70.5%

C Cell barcode mapping statistics

Total_pairs	105219588	100%
Total_pairs_mapped	105219588	100%
Total_uniq_mapped	105219352	100%
Total_mito_mapped	0	0%
Total_dups	2005748	1.9%
Total_pairs_MAPQ30	94743381	90%
Total_mito_MAPQ30	0	0%
Total_dups_MAPQ30	1300676	1.2%
Library complexity		100%

Fig S6. Quality assessment metrics for called single cells of Buenrostro 2018 data set. (**A**) Plot of the fraction of fragments in peaks versus the total number of unique fragments. The plot can be used to distinguish cell barcodes from non-cell barcodes. (**B**) Distribution of insert fragment sizes. The plot can be used to evaluate the quality of transposase reaction. (**C**) Transcription start site (TSS) enrichment profile. (**D**) Distribution of the total number of unique fragments for cell and non-cell barcodes. The plot can be used to evaluate the amount of cell debris sequenced. (**E**) Boxplot of fragments overlapping annotated genomic regions per cell. (**F**) Overall statistics of data aggregated from all called cells.

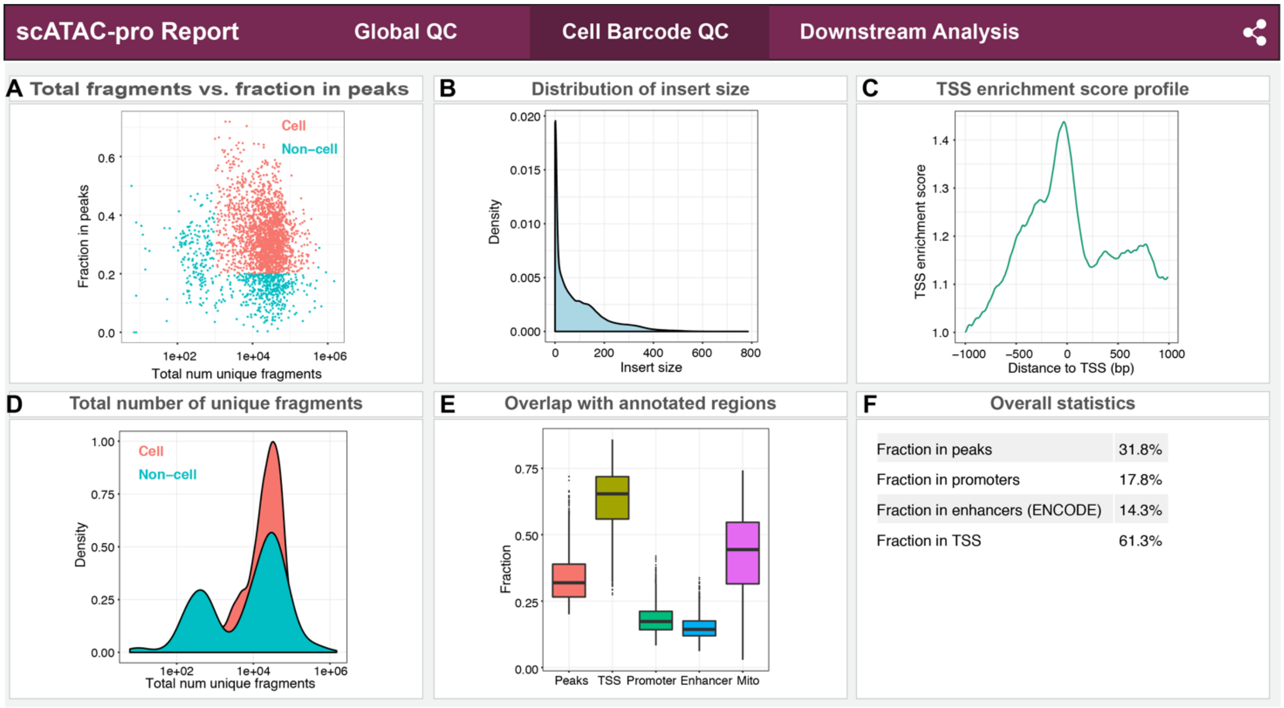

Fig S7. Quality assessment metrics for called single cells of Cusanovich 2018 data set. (**A**) Plot of the fraction of fragments in peaks versus the total number of unique fragments. The plot can be used to distinguish cell barcodes from non-cell barcodes. (**B**) Distribution of insert fragment sizes. The plot can be used to evaluate the quality of transposase reaction. (**C**) Transcription start site (TSS) enrichment profile. (**D**) Distribution of the total number of unique fragments for cell and non-cell barcodes. The plot can be used to evaluate the amount of cell debris sequenced. (**E**) Boxplot of fragments overlapping annotated genomic regions per cell. (**F**) Overall statistics of data aggregated from all called cells.

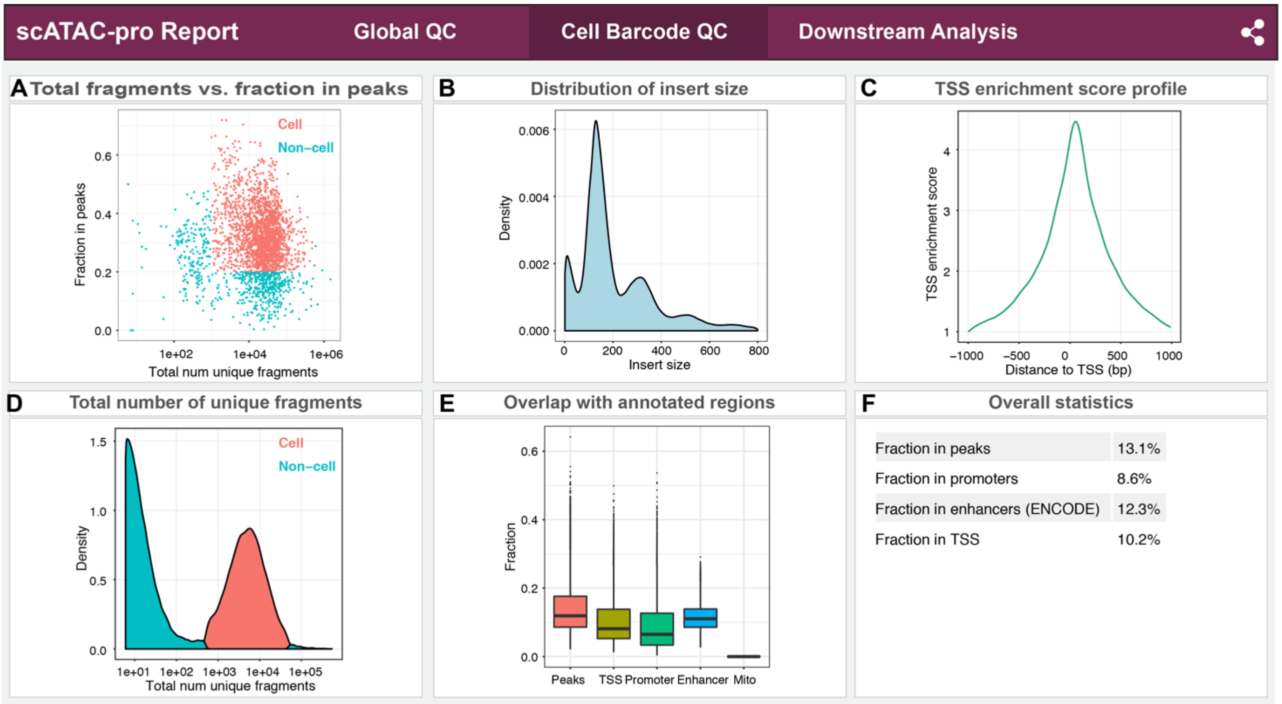

Fig S8. Summary report for downstream analyses of Buenrostro 2018 data set. Results of the following analyses are shown: clustering analysis (**A**), transcription factor (TF) motif enrichment analysis (**B**), differential footprinting analysis (between one cluster and the rest of the clusters (**C**), enriched gene ontology (GO) terms for cluster0 (**D**), and predicted cis-interactions at *GATA1* locus (**E**).

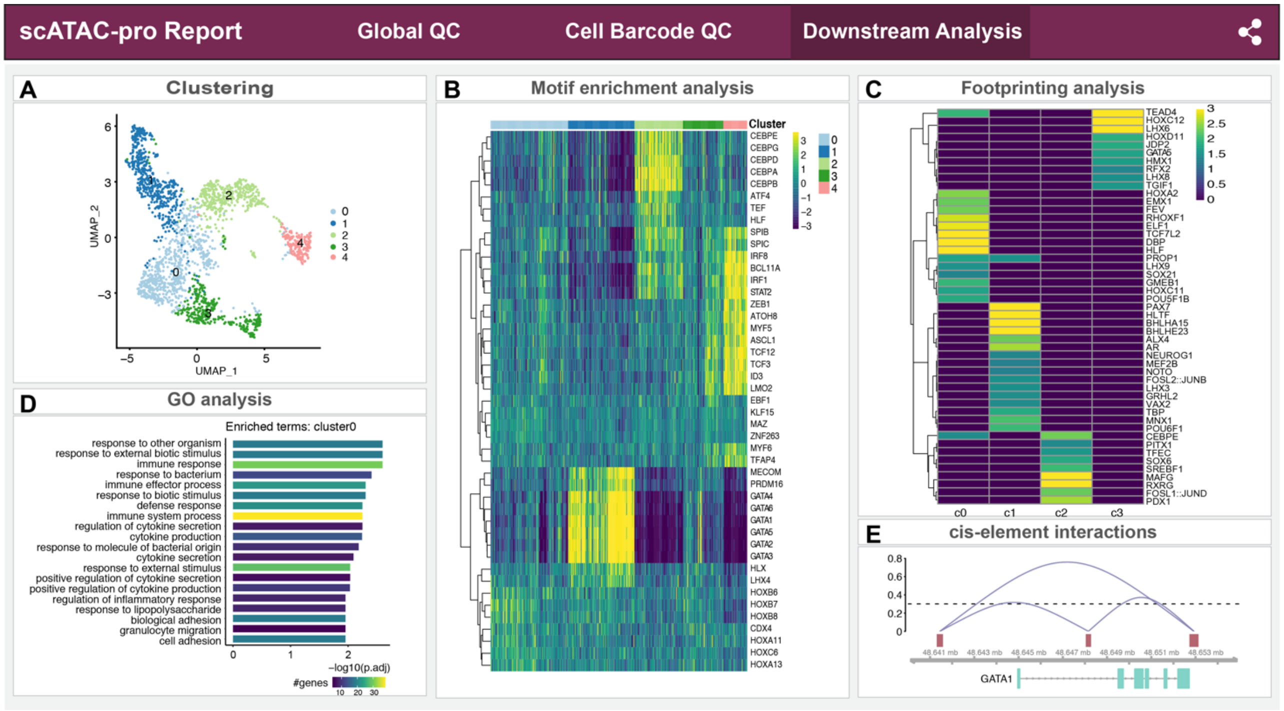

Fig S9. Summary report for downstream analyses of Cusanovich 2018 data set. Results of the following analyses are shown: clustering analysis (**A**), transcription factor (TF) motif enrichment analysis (**B**), differential footprinting analysis (between one cluster and the rest of the clusters) (**C**), enriched gene ontology (GO) terms for cluster8 (**D**), and predicted cis-interactions at *CEBPB* locus (**E**).

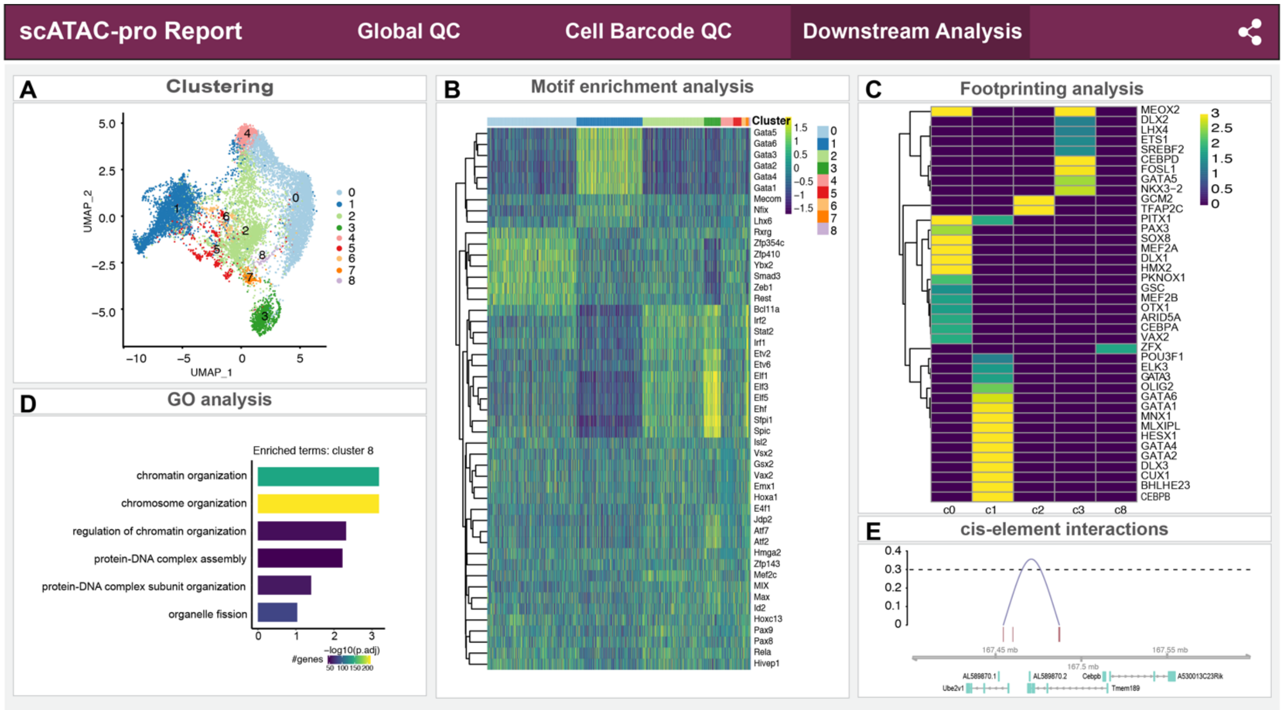

Fig S10. Screenshot of the user interface of the visualization tool, *VisCello.* scATAC-Seq data of human peripheral blood mononuclear cells (PBMCs) was used for illustration purpose. (**A**) Chromatin accessibility score of peak overlapping with the transcriptional start site of *MS4A1* is displayed. Users can use gene name or peak coordinate as the search keyword to explore the accessibility of interested regions. The raw and normalized data can be visualized using uniform manifold approximation and projection (UMAP) or t-distributed stochastic neighbor embedding (tSNE) with different numbers of principal components. (**B**) Differential chromatin accessibility analysis. The comparison can be done between any two groups of cells specified by the user. The resulting set of differential accessible regions and the heatmap are downloadable. Shown is the comparison between monocytic cell clusters (clusters 0, 6, 7, 8) versus T cell clusters (clusters 1, 2, 5).

A

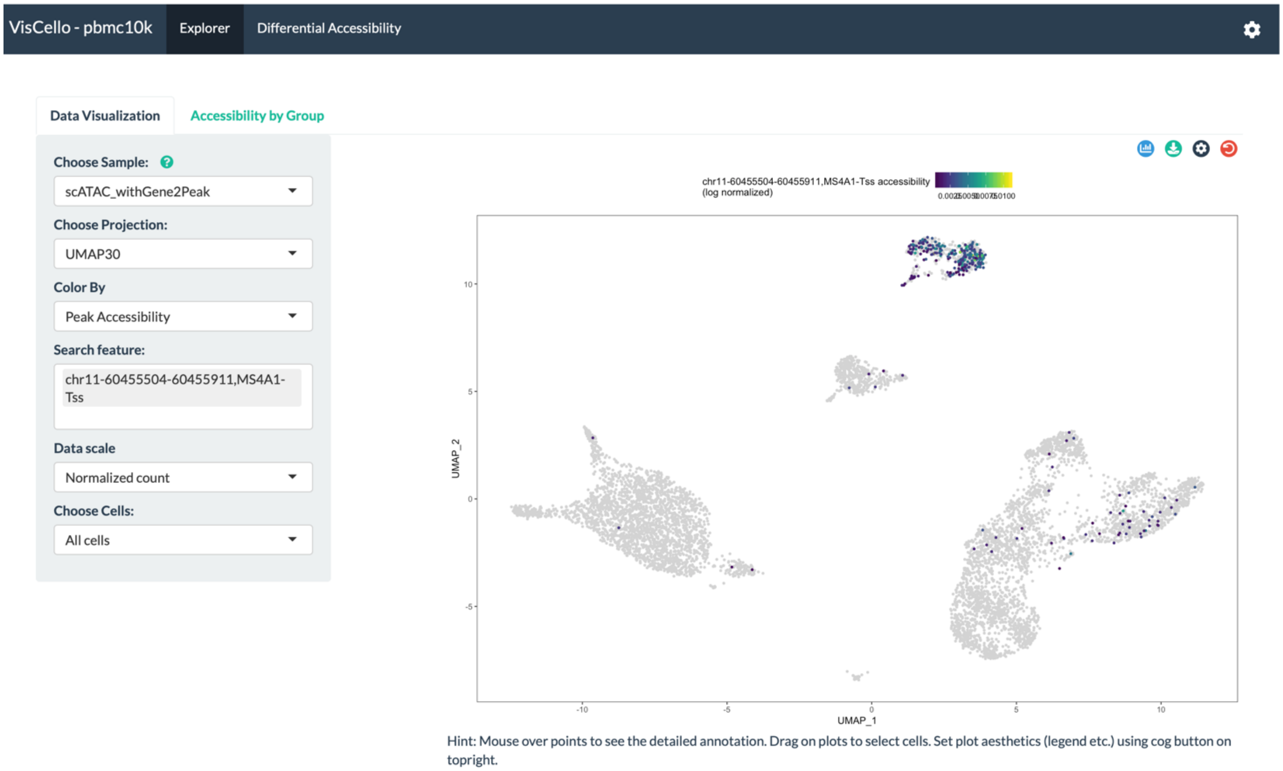
VisCello - pbmc10k
Explorer
Differential Accessibility
Data Visualization
Accessibility by Group
Choose Sample:
scATAC_withGene2Peak
Choose Projection:
UMAP30
Color By
Peak Accessibility
Search feature:
chr11-60455504-60455911,MS4A1-Tss
Data scale
Normalized count
Choose Cells:
All cells
chr11-60455504-60455911,MS4A1-Tss accessibility
(log normalized)
UMAP_2
UMAP_1
Hint: Mouse over points to see the detailed annotation. Drag on plots to select cells. Set plot aesthetics (legend etc.) using cog button on topright.

B

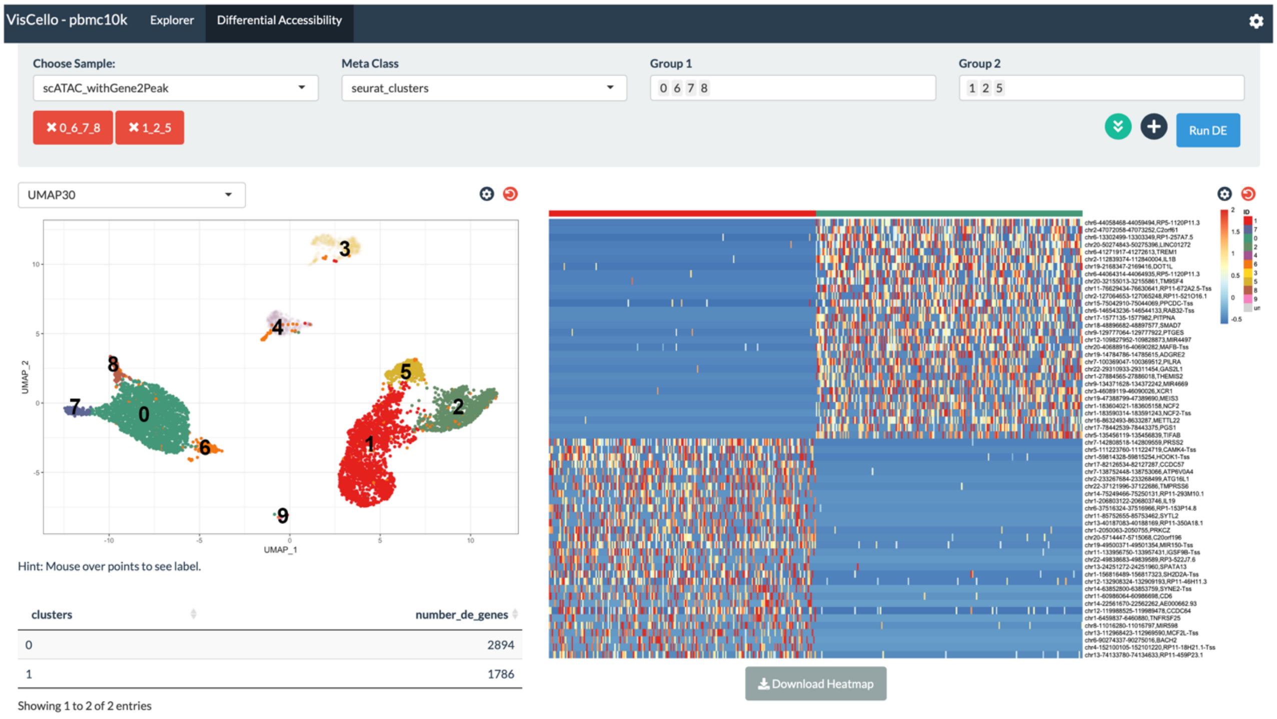
VisCello - pbmc10k
Explorer
Differential Accessibility
Choose Sample:
scATAC_withGene2Peak
Meta Class
seurat_clusters
Group 1
0 6 7 8
Group 2
1 2 5
0_6_7_8
1_2_5
Run DE
UMAP30
UMAP_2
UMAP_1
Hint: Mouse over points to see label.
clusters
number_de_genes
0
2894
1
1786
Showing 1 to 2 of 2 entries
Download Heatmap

Fig S11. Chromatin accessibility of transcription start site (TSS) of two dendritic cell markers *CST3* (A) and *FCER1A* (B) shown in UMAP and UCSC genome browser, respectively.

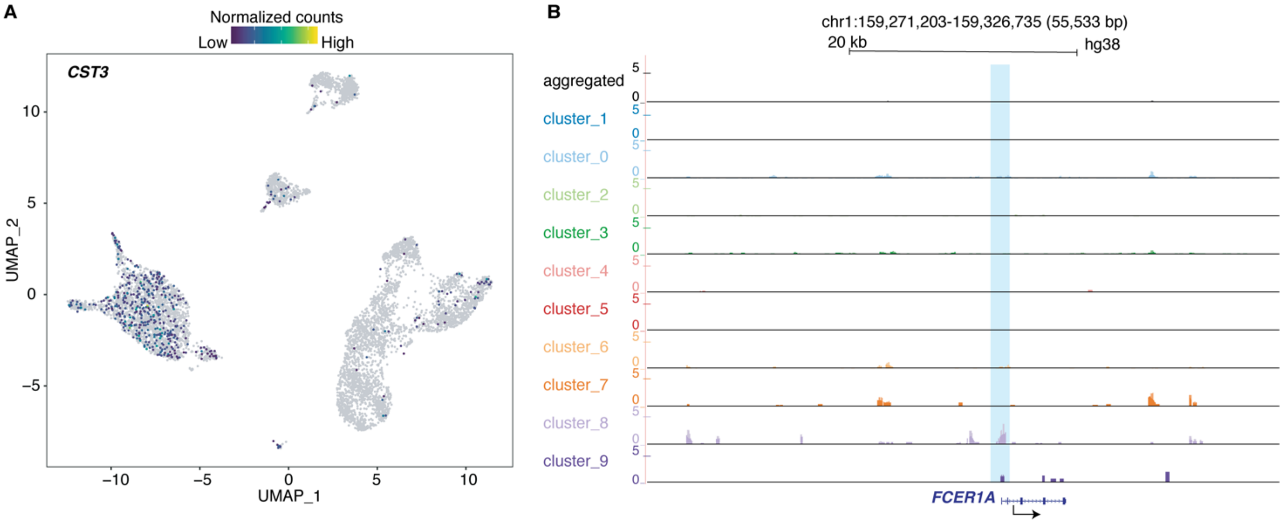

Supplement: Supplementary file 1 — Additional file 1: Figs. S1–11. [file 13059_2020_2008_MOESM1_ESM.pdf]
