# Supplementary material for: scATAC-pro: a comprehensive workbench for single-cell chromatin accessibility sequencing data
Source: Genome Biol. 2020 Apr 20;21:94. doi: 10.1186/s13059-020-02008-0 (PMC7169039; doi:10.1186/s13059-020-02008-0)
Supplement: Supplementary file 2 — Additional file 2: Table S1. [file 13059_2020_2008_MOESM2_ESM.pdf]

Supplementary Tables

Table S1. Comparison of the features of three comprehensive software for processing and analysis of single-cell chromatin accessibility sequencing data. MDS, multidimensional scaling; NB, negative binomial; PLSA, probability latent semantic analysis; LSI, latent semantic indexing; LDA, latent Dirichlet allocation; UMAP, uniform manifold approximation and projection (UMAP); tSNE, t-distributed stochastic neighbor embedding.

	**Scasat**	**cellranger-atac**	**scATAC-pro**
Open source	Yes	No	Yes
Preprocessing	Adapter trimming	Demultiplexing, adapter trimming	Demultiplexing, adapter trimming
Read mapping	Bowtie2	BWA	BWA, Bowtie, Bowtie2
Peak calling	MACS2	ZINBA [48]	MACS2, GEM, BIN, COMBINED
Cell calling	Filtering	Mixture of NB model	Filtering, EmptyDrop, Mixture of NB model
Dimension reduction	MDS, tSNE	PCA, LSI, PLSA, tSNE	PCA, LDA, LSI, tSNE, UMAP
Clustering	k-medoids on original features	K-means, Louvain on features of reduced dimensions	Louvain, K-means on features of reduced dimensions, and cisTopic
TF motif enrichment analysis	No	Custom code	chromVAR,
TF footprinting analysis	No	No	HINT
Differential accessibility analysis	Information gain, Fisher's exact test	Custom model	DESeq2, Wilcoxon test, logistic regression, negative binomial test
GO analysis	Yes	No	ClusterProfiler
Summary report	No	HTML	HTML
Generation of Genome browser track files	Yes	No	Yes
Support of multiple experimental protocols	Yes	No	Yes
Cis-chromatin interactions	No	No	Cicero
Integration of multiple scCAS data sets	No	No	Seurat v3, Harmony, Pool
Visualization	No	No	VisCello
